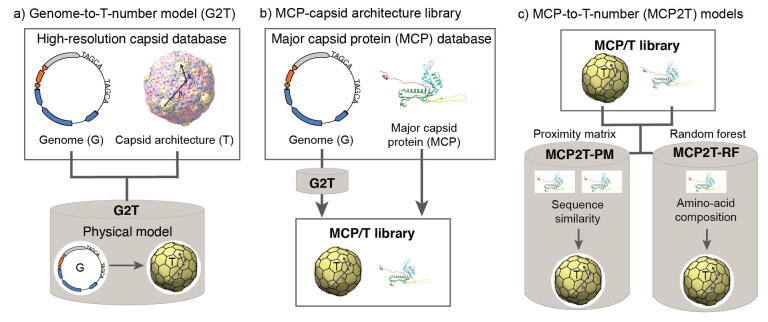# Predicting the capsid architecture of phages from metagenomic data

**DOI:** 10.1016/j.csbj.2021.12.032

**Published:** 2022-01-05

**Authors:** Diana Y. Lee, Caitlin Bartels, Katelyn McNair, Robert A. Edwards, Manal A. Swairjo, Antoni Luque

**Affiliations:** a Viral Information Institute, San Diego State University, 5500 Campanile Drive, San Diego, CA 92182, USA; b Computational Science Research Center, San Diego State University, 5500 Campanile Drive, San Diego, CA 92182, USA; c Department of Biology, San Diego State University, 5500 Campanile Drive, San Diego, CA 92182, USA; d Flinders Accelerator for Microbiome Exploration, Flinders University, Bedford Park, GPO Box 2100, Adelaide 5001, South Australia, Australia; e Department of Chemistry and Biochemistry, San Diego State University, 5500 Campanile Drive, San Diego, CA 92182, USA; f Department of Mathematics & Statistics, San Diego State University, 5500 Campanile Drive, San Diego, CA 92182, USA

**Keywords:** Tailed bacteriophages, Icosahedral capsids, Physical modeling, Machine learning, Metagenomes, Gut microbiome, Viral ecology, Physical virology

## Abstract

Tailed phages are viruses that infect bacteria and are the most abundant biological entities on Earth. Their ecological, evolutionary, and biogeochemical roles in the planet stem from their genomic diversity. Known tailed phage genomes range from 10 to 735 kilobase pairs thanks to the size variability of the protective protein capsids that store them. However, the role of tailed phage capsids’ diversity in ecosystems is unclear. A fundamental gap is the difficulty of associating genomic information with viral capsids in the environment. To address this problem, here, we introduce a computational approach to predict the capsid architecture (T-number) of tailed phages using the sequence of a single gene—the major capsid protein. This approach relies on an allometric model that relates the genome length and capsid architecture of tailed phages. This allometric model was applied to isolated phage genomes to generate a library that associated major capsid proteins and putative capsid architectures. This library was used to train machine learning methods, and the most computationally scalable model investigated (random forest) was applied to human gut metagenomes. Compared to isolated phages, the analysis of gut data reveals a large abundance of mid-sized (T = 7) capsids, as expected, followed by a relatively large frequency of jumbo-like tailed phage capsids (T ≥ 25) and small capsids (T = 4) that have been under-sampled. We discussed how to increase the method’s accuracy and how to extend the approach to other viruses. The computational pipeline introduced here opens the doors to monitor the ongoing evolution and selection of viral capsids across ecosystems.

## Graphical abstract

**Figure:**
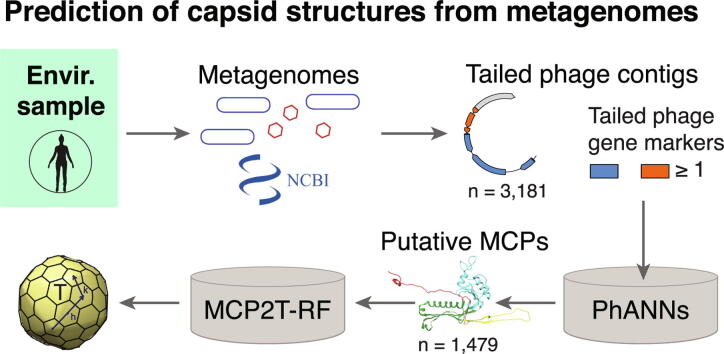

## 1 Introduction

Tailed phages are viruses that infect bacteria and have evolved an extremely diverse set of protein capsid architectures to protect their infective genome [82], [12]. Tailed phage capsids sizes range from 40 nm to 180 nm in diameter [95], [118], [51]. The internal volumes of these capsids accommodate genomes spanning three orders of magnitude in length, from 5 kilobase pairs (kbp) to 735 kbp [82], [62]. The diversity in genome length and genomic content of tailed phages may explain their key role in regulating ecosystems [86], [113], in promoting the evolution of microbes [129], [67], [121], in participating strongly in the planetary carbon cycle [74], and in becoming the most abundant biological entity on the planet [27]. However, the role of the diversity in tailed phage capsid architectures and genome lengths across ecosystems remains unclear.

A key challenge investigating the selection and evolution of tailed phage capsids is linking viral capsids with their viral genome in the environment [19]. The number of phages isolated and studied both genetically and structurally [34], [70] represent a very small sample compared to the number of viruses evolving in the environment [27], [6], [29], [38], [109], [50], [103], [11], [107]. Electron microscopy can measure the morphology and size of tailed phages, but these observations do not include genomic information, limiting how to interpret the change in capsid size distributions observed across ecosystems[119], [18]. There are trade-offs in selecting capsid sizes that are difficult to disentangle [37]. An increase in temperature may promote smaller genomes among viruses and other organisms [90], but larger genomes encode more genes, which can enhance the survival of both phages and their hosts[120], [110], [112]. On the other hand, larger genomes and their associated larger capsids are more costly energetically, which can compromise their replication in limiting growth conditions [20], [84]. Additionally, an increase in size reduces virus diffusivity [26], which can negatively impact their infectivity [66]. To link the capsid and genomic information of viruses in the environment, we introduced a new computational approach that builds on the established geometrical principles governing the capsid structure and genome packing of tailed phages [102], [81], [44], [118], [42], [82].

**Fig. 1:**
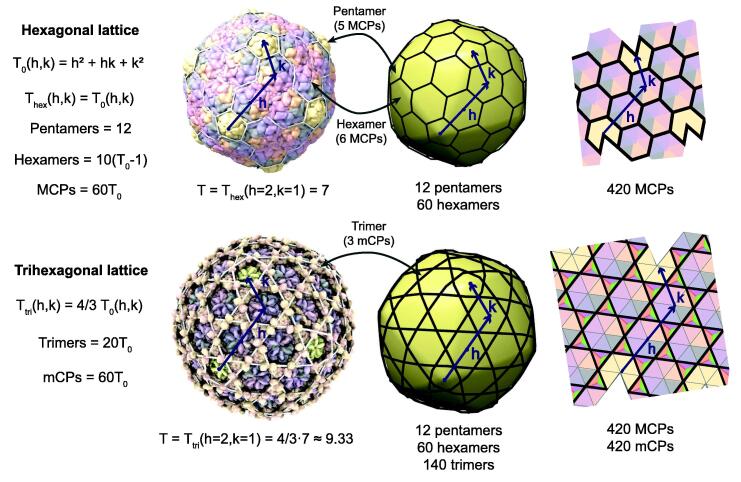
**Icosahedral capsids among tailed phages.** The hexagonal (top) and trihexagonal (bottom) icosahedral lattices observed among icosahedral tailed phage capsids. In both lattices, major capsid proteins (MCPs) form clusters (capsomers) of five (pentamers) and six (hexamers) proteins. Two nearby pentamers are connected by *h* and *k* steps crossing over hexamers. The trihexagonal lattice also contains minor capsid proteins (mCPs) clustered in groups of three (trimers). The T-number is proportional to the number of major and minor capsid proteins. T_0_ is the T-number defined by the classic icosahedral capsid theory [24]. T_hex_ and T_tri_ are the T-numbers associated, respectively, with the hexagonal and trihexagonal lattices defined by the generalized icosahedral capsid theory[122]. The top and bottom capsid examples correspond, respectively, to phage HK97 (PDB 2 fs3; [47]and phage patience (EMDB-21123; [99]. The capsids were rendered with ChimeraX[96]. The 3D icosahedral lattice models were produced with the generalized *hkcage* tool in ChimeraX[82].

The majority—80% to 90%—of tailed phage capsids are quasi-spherical [2], [3]. The remaining tailed phages adopt elongated capsids with icosahedral caps [3], [80]. Among tailed phages, the capsids are built from multiple copies of the major capsid protein, which systematically adopt the HK97-fold [124], [98], [34]. Capsid proteins in tailed phages are organized following hexagonal and trihexagonal icosahedral lattices, Fig. 1 [122], [99], [82], and the double-stranded DNA genome is packed in the capsid at quasicrystalline densities [36], [78], [82]. The number of capsid proteins is determined by the triangulation number or T-number, which is a discrete index determining the possible capsid surfaces compatible with icosahedral symmetry [24], [122]. The number of major capsid proteins is 60 T_0_ (Fig. 1), where T_0_ represents the classic T-number:

(1) $$T_{0}\left(h,k\right)=h^{2}+hk+k^{2}\;\;.$$

In the generalized theory for icosahedral capsids, the T-number for the hexagonal lattice is T_hex_ = T_0_, and the T-number for the trihexagonal lattice is T_tri_ = 4/3 T_0_ [122]. The factor 4/3 ≈ 1.33 accounts for the additional surface associated with 60 T_0_ minor capsid proteins inserted as trimers in the trihexagonal lattice (Fig. 1). Experimental and bioinformatic studies indicate that tailed phages can adopt capsid architectures from putative T = 1.33 capsids to T = 52 capsids [118], [82]. The T-number follows an allometric relationship with the genome length with an approximate exponent of 2/3 ≈ 0.67 because the T-number is proportional to the capsid surface and the genome length is proportional to the capsid volume [82]. Thus, the increase in genomic content is associated with larger tailed phage capsids built with more capsid proteins. Since the major capsid proteins conserve the HK97-fold while adopting a large diversity of sequences, here we propose that part of this sequence diversity is associated with the formation of different T-number capsids.

**Fig. 2:**
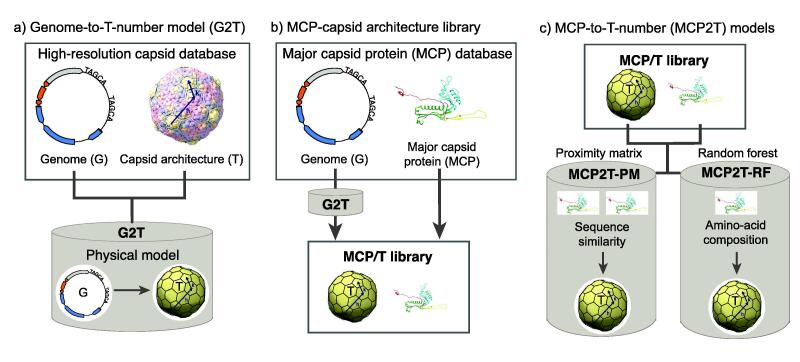
**Computational approach to predict capsid architecture from genomic information.** a) A database containing tailed phage genomes and their associated high-resolution capsid reconstructions was used to validate the physical genome-to-T-number (G2T) model. b) A database containing isolated tailed phage genomes and encoded HK97-fold major capsid proteins (MCPs) was curated. The G2T model was applied to identify the putative T-number capsid architectures associated with each HK97-fold MCP, obtaining the MCP/T library. c) The MCP/T library was used to train statistical learning methods to predict the capsid architecture of tailed phages from information in the MCP sequence, leading to the major capsid protein-to-T-number (MCP2T) models. The MCP2T-PM model was built on a proximity matrix (PM) algorithm using protein sequence similarity. The MCP2T-RF model was built on a random forest algorithm using MCP amino-acid composition as features.

Confirming a direct relationship between major capsid protein sequences and T-number capsids would open the doors to predicting the capsid architecture of tailed phages (and genome lengths) from a single gene. This would facilitate inferring tailed phage capsids from sequenced environmental data that is now obtained routinely [17], [112], [103], [77], [105]. To test the capsid protein-to-T-number association, we developed a computational approach that can predict accurately the capsid architectures of tailed phages from the major capsid protein gene (Fig. 2). First, the genome-to-T-number model (G2T) was extended by training a power function physical model using a larger database of high-resolution tailed phage capsids than prior studies (Fig. 2a). Major capsid proteins (MCPs) adopting HK97-fold were obtained from tailed phage genome isolates, and the G2T model was applied to the genomes to obtain the putative capsid architectures among these phage isolates, generating the MCP/T library (Fig. 2b). The MCP/T library was used to train the major capsid protein-to-T-number (MCP2T) models using a proximity matrix approach (MCP2T-PM) and a random forest approach (MCP2T-RF) (Fig. 2c). Finally, these statistical learning models were applied to metagenomic data to infer the capsid architecture of uncultured tailed phages in the human gut.

## 2 Methods

The GitHub repository http://github.com/Luquelab/Lee_etal_CSBJ_2022 contains the codes and instructions necessary to implement the methods and replicate the research. The supplementary section SI-1 contains the description of the supplementary Data Files referenced in the Methods and Results sections.

**Genome-to-T-number (G2T) model.** The genome-to-T-number (G2T) model is a physical model that predicts the capsid architecture (T-number) of a tailed phage from its genome length (Fig. 2a). The G2T model was introduced in [82]. The model relies on the empirically and theoretically justified physical allometric relation between the genome length and capsid architecture of tailed phages [82]. Here, the G2T model was revised, increasing the database of high-resolution structure to train and test the model (from 23 to 37 structures) as detailed in the *Data acquisition* section below. Another novelty was the error analysis of the model and error prediction when increasing the training data set, as detailed in the *Model accuracy* section below.

*Data acquisition.* Tailed phages containing high-resolution capsid data were initially identified from a review article in the field [118], the icosahedral capsid database VIPERdb [89], and four recently reconstructed tailed phages displaying new T-numbers: the jumbo tailed phage SCTP2 [59] and P74-26, P23-45, and Mic1 [116], [8], [65]. The capsid protein stoichiometry and high-resolution structures were revised to update the T-numbers according to the generalized quasi-equivalence icosahedral framework, including hexagonal and trihexagonal lattices observed among tailed phages [122]. The final high-resolution database included n_HR_ = 37 tailed phage capsid structures (Table 1 and Data File 1).

**Table 1:** High-resolution capsid database. See additional information in Data File 1.

**Phage**	**T**	**Genome (kbp)**	**Reference**
C1	4	16.7	[5]
HSTV-1	7	32.2	[98]
P2	7	33.6	[31]
TP901-1	7	37.7	[9]
Sf6	7	39.0	[92]
ε15	7	39.7	[7], [64]
HK97	7	39.7	[48], [55], [124]
T7	7	39.9	[4], [53], [61]
CUS-3	7	40.2	[93]
HK022	7	40.8	[100]
Pf-WMP4	7	40.9	[132]
BPP-1	7	42.9	[127]
P22	7	43.5	[25], [91]
80α	7	43.9	[115]
K1E/K1-5	7	44.7	[75]
P-SSP7	7	45 . 0	[79]
Gifsy-2	7	45.8	[40]
Syn5	7	46.2	[49], [131]
Λ	7	48.5	[73]
CW02	7	49. 4	[108]
SPP1	7	49.5	[123]
SIO-2	12	80.0	[72]
P74-26	9.33	83.0	[116]
P23-45	9.33	84.2	[8]
Basilisk	12	81. 8	[52], [122]
Mic1	13	92.6	[65]
T5	13	121. 8	[39]
SPO1	16	132. 6	[35]
ΦM12	19	194.7	[117]
N3	19	207.0	[118], [59]
PAU	25	219.0	[118], [59]
ΦRSL1	27	240.0	[41]
PBS1	27	252.0	[118], [59]
ΦKZ	27	280.0	[45]
121Q	28	348.5	[118]
SCTP2	39	440.0	[59]
G	52	498.0	[118], [59]

*Statistical model.* A power function model $T\left(G\right)={b\left(\frac{G}{G_{0}}\right)}^{a}$ related the T-number as a function of the genome length, *G*. Here, *b* was the prefactor constant, *a* the allometric exponent, and *G_0_* the reference units of G, *G_0_* = 1 kbp. This allometric relationship was empirically and theoretically established previously for a smaller number of tailed phages [60], [82]. The allometric relationship is a consequence of the constant density of the genome stored in tailed phage capsids and constant surface of the major protein on the capsid exterior [82]. The theory predicts an allometric exponent *a_th_ = 2/3* because the T-number scales like the capsid surface and the genome scales with the capsid volume. A derivation of the theoretical prediction is provided in the supplementary section SI-2. The model was linearized using a logarithmic transformation:

(2) $$\ln(T)=a\;ln(G/G_{0})+ln(b)\;\;.$$

The slope, *a*, and intercept, ln *b*, of best fit were obtained using the least squares method in the *Linear Regression* function from the Scikit learn package for Python [94]. The residual bias and coefficient of determination of this model were compared with alternative models (exponential, quadratic, reciprocal, logarithmic) for quality control, confirming the adequacy of the power function model (see supplementary section SI-3 and Fig. S1).

*Model accuracy.* The accuracy of the G2T model was investigated statistically using different training sets. This estimated the expected model’s error and facilitated making projections to judge if increasing the data set would improve the model. The approach was as follows. The best fit values for the G2T model, Eq. (2), were obtained using different training data sets of size n, ranging from *n* = 5 to n = 30. The n data points in a training data set were chosen randomly from the high-resolution tailed phage capsid database (Table 1). For each model, the T-number was predicted from the genome length of the remaining capsid structures (n_HR_ – n, that is, 37 – n). The relative error was defined as the model’s residual (difference between the predicted T-number and the empirical T-number) divided by the empirical T-number. This process was repeated 10,000 times for each n to estimate the G2T’s mean relative error (MRE) as a function of the training data set size, n. To predict the accuracy of the model for data sets larger than the current database, (n > n_HR_), the mean relative error was fitted to the exponential model

(3) $$\mathrm{MRE}\left(n\right)={\mathrm{pe}}^{-qn}+w\;.$$

The values of best fit for the parameters *p*, *q*, and *w* were obtained applying the robust least squares method from the least squares function in the Python’s SciPy optimize package [125]. The confidence interval of the parameters was estimated by bootstrapping 10,000 random subsets and fitting Eq. (3) in each case. A genome length was associated with a T-number in the hexagonal or trihexagonal lattice if the uncertainty of the predicted T value, that is, T ± ΔT, contained such T-number. The uncertainty ΔT was calculated based on the mean relative error projected from Eq. (3) for the size of the high-resolution database, n = n_HR_ = 37, that is, ΔT = T·MRE(n_HR_).

**MCP/T library.** Major capsid protein amino acid sequences associated with tailed phages were obtained from isolated genomes accessed on the phantome.org website in January 2017 [87], [97]. Genomes listed as *Caudovirales* (the taxonomic order of tailed phages) in the GenBank *ORGANISM* field were filtered. Among the 2,996 *Caudovirales* genomes identified, protein-coding genes (CDS) containing the term “major capsid” as a product keyword were selected, leading to 669 putative tailed phage major capsid proteins. The folded structures for the selected major capsid proteins were obtained investigating structural relatives in HHpred using the PDB database and submitting the top candidates (above 95% probability) to Modeller [128], [46], [114], [56], [88]. The folded models were inspected visually. Only those major capsid proteins displaying the canonical features of the HK97-fold were selected [118]. Major capsid proteins identified in phage genomes from the high-resolution capsid database were also included. The protein sequences associated with open reading frames (ORFs) in these genomes were retrieved from NCBI. Structural functions were identified from the protein sequences using the Phage Artificial Neural Networks (PhANNs) web server [21]. Sequences predicted to be major capsid protein as the most likely function and displaying a score ≥ 2 (98% true positive confidence) were selected. The HK97-fold in these proteins was validated combining HHpred and Modeller as described above. HK97-fold MCP proteins were obtained for 31 out of 37 phages in the high-resolution database. The exceptions were Gifsy-2, SIO-2, Basilisk, ΦRSL1, ΦKZ, and SCTP2. This led to a final library of *n_lib_* = 635 HK97-fold MCPs associated with tailed phage genome lengths (Data File 2 and Fig. 2b).

The distribution of genome lengths was investigated using the non-parametric kernel density estimation method. To capture accurately the multimodal nature of the phage genome length distribution, the kernel bandwidth was investigated independently for four distinctive genome length groups identified visually: 17–130 kbp, 130–210 kbp, 210–270 kbp, and 270–498 kbp (Supplementary Fig. S2). The Scikit grid search 5-fold cross-validation method [94] was applied to obtain the most likely Gaussian kernel’s bandwidth for each group, leading to 1.78 kbp, 3.33 kbp, 1.39 kbp, and 20 kbp, respectively. The four distributions were combined and normalized to obtain a single probability density function of tailed phage genome lengths. The peaks of the distribution were obtained using the find peaks function from the SciPy signal package for Python [125].

The library containing MCPs and the associated T-numbers (MCP/T library) was built as follows (Fig. 2b). For the 31 MCPs found in the high-resolution capsid database, the T-number used in the library was the one associated with the 3D capsid architecture. For the rest of the isolated tailed phage genomes, the T-number was predicted applying the G2T model to the genome length. If the predicted T-number fell within the ranges of one or several overlapping T-numbers regions, the T-number selected was closest to the mean predicted T-number, and the alternative T-numbers were tallied. For T-numbers associated with multiple lattices (for example, T = 12 trihexagonal versus T = 12 hexagonal), each architecture was considered as a potential structure. If the predicted T-number was not within the error margin of a valid icosahedral T-number, the architecture was categorized as “elongated.”

**MCP-to-capsid model based on similarity (proximity matrix): MCP2C-PM.** Protein-protein sequence similarities were obtained for the MCPs in the library using NCBI blastp [13], [85], applying the default algorithm parameters except for the e-value threshold, which was chosen to be 0.001 to increase the quality and decrease the effects of randomness for the matches. In any instance where blastp returned more than one score for any pair of phages, the higher similarity score was chosen for the pair. In the MCP/T library, 80% of the data was selected randomly as the training set and the remaining 20% was used as the test dataset (80/20 split). For statistical robustness, 1000 different 80/20 training and test splits were generated. For each major capsid protein sequence in the test set, the T-number predicted corresponded to the T-number associated with the most similar major capsid protein sequence in the training set (proximity matrix). A prediction was considered correct if the T-number predicted coincided with the T-number associated with the major capsid protein in the MCP/T library. The model accuracy was defined as the fraction of correct predictions in the full test dataset. The accuracy was investigated as a function of different minimum similarity thresholds, from 0% to 100% similarity in increments of 10%. In each case, the fraction of predicted architectures was tallied.

*MCP phylogenetic tree.* The protein sequences of the MCPs in the MCP/T library were aligned using the Clustal Omega webserver (default settings) at https://www.ebi.ac.uk/Tools/msa/clustalo/ [111]. The resulting phylogenetic tree (ClustalW format) was visualized and analyzed using the Interactive Tree of Life (iToL) webserver at https://itol.embl.de [76]. Each MCP in the tree included the associated phage genome length (using the log-linear transformation G_t_ = log_10_(genome length in kbp)-3) and the capsid architecture predicted by the G2T model. Clades displaying common properties were identified qualitatively from the tree internal structure and phage phenotypic data.

**MCP-to-capsid model based on random forest: MCP2C-RF.** The similarity model introduced above has two important limitations. First, the method cannot predict the capsid architecture for major capsid proteins that have no similarity in the MCP/T library. This is a bottleneck for environmental analysis because most uncultured tailed phage genes have little similarity to genes in public databases [130], [71]. Second, the matrix similarity is a computational search method of quadratic order, *O(n_lib_^2^)*, which limits the scalability of the model when increasing the size of the training library, *n_lib_*. To circumvent these foreseeable challenges when characterizing environmental data, an alternative machine learning method was investigated and compared. The approach chosen was random forest because it offers a rapid learning process when the training sets are small with respect to the dimensionality of data, and the cost of prediction is independent of the training data set’s size [63].

Random forest regression is an ensemble statistical learning algorithm that generates multiple decision trees using a collection of input features as nodes and the value of the dependent variable (output) at the end of the node. To create each of these decision trees, *m* random observations and *f* random features are selected from the original data and the corresponding labels used as targets. A final sorting decision is made based on the trees formed by the training data and can then be used to generate a proposed label for each test data point [57], [16]. A total of 22 MCP features were used to train the random forest model: protein sequence length, the protein’s isoelectric point, and the frequency of each amino acid in the MCP sequence (20 features referred to as the amino acid composition). The isoelectric point was calculated using Biopython’s sequence utility package [28]. These protein features have been previously used to identify functions of viral proteins efficiently in machine learning approaches [106], [21]. The T-number associated with each major capsid protein in the MCP/T library was used as the label for the random forest classification. T-numbers that were overlapping based on the confidence interval of the G2T model were combined in single classes. Due to the small density of large genomes, architectures T ≥ 25 were groped as a single class. An 80/20 training/test split was applied to the library to test the random forest model. The random forest parameters were optimized for accuracy using Scikit’s GridSearchCV function [94] using 80% of the library. The top 10 estimators were run 100 times each to verify the aggregate highest average accuracy. This led to a maximum number of 4 features per tree, 250 estimators, a max depth of 20, 1 minimum sample in a leaf, and a minimum sample split of 46, with data bootstrapping, and using a balanced weight distribution. To ensure statistical robustness, the random forest model was then tested selecting 1000 different randomly generated training datasets from the MCP/T library. Given a major capsid protein sequence, a predicted capsid architecture was considered correct if the predicted T-number was within the margin of error (9%) expected associated with the T-number in the MCP/T library. The number of correctly predicted phages was tallied and used to calculate a percentage accuracy for that test set. Both permutation and dropout analysis were performed on all features. The randomization or omission of no single feature caused deviation greater than 8 %. To gain insight in the interpretation of the random forest model, the 22 features of the model were analyzed for the main clades identified in the phylogenetic analysis and compared to the average features in the MCP/T library. Those features departing on average more than a standard deviation from the reference value were identified as significant.

To determine the impact of increasing the training library in the accuracy of the random forest model, the accuracy of the model was assessed for different library sizes and fitted to a mathematical model. The different sizes for the training set were defined as n_i_ = n_lib_ i/20 for a total of twenty training sizes, *i* = 1 to 19. The size of the testing set was n_lib_ – n_i_ = n_lib_ (1– i/20). For statistical robustness, 1000 different training sets were generated for each size n_i_ and the mean accuracy was measured in each case, MACC_i_ . The mean accuracy values were fitted to the logarithmic model

(4) $$\mathrm{MACC}\left(n\right)=g\log_{10}n+h\;\;.$$

The values of best fit for the parameters *g* and *h* were obtained using the robust least squares method. The confidence intervals of the values of best fit were obtained by bootstrapping 10,000 subsets generated randomly from the estimated mean accuracies, *MACC_i_*.

**Computational performance of MCP2C models.** The computational scalability of the proximity matrix similarity (MCP2C-PM) and random forest (MCP2C-RF) models was estimated generating larger artificial libraries. The original MCP/T library (*n_lib_* = 617) was sequentially used 15 times, generating 15 artificial libraries with 617 to 10,035 entries. Both models were trained (80/20 split) for 100 different randomly selected training sets for each library size. For each training, the elapsed training time was recorded, and the statistics of the training time were obtained for each model and library size. Then, the T-number of 50 major capsid protein sequences were predicted to tally in each case the elapsed time for the prediction. These time-searches were averaged for each generated model and library size. Linear and quadratic models were fitted to the average times as a function of the library size using least-squares method via numpy polyfit [54]. These fitted models were used to extrapolate the scalability of the two methods for libraries as large as 1,000,000 entries. The elapsed times were obtained on Lenovo laptop with an intel i7 processor and 16 GB RAM.

**Capsid architecture prediction from gut metagenomes.** 3,173 metagenomically assembled circular genomes (direct terminal repeats ≥ 50 bp) and at least two canonical tailed phage markers published in [11] were accessed at ftp://ftp.ncbi.nih.gov/pub/yutinn/benler_2020/gut_phages/ in the NCBI server. The open reading frame sequences (putative proteins) were input to the PhANNs web server [21]. Proteins that displayed major capsid protein function as the highest score were selected. Those proteins with score ≥ 2 were further selected (expected accuracy—true positives—of using this score is 98%). When the same MCP was found in similar metagenomic assembled genomes, one representative was kept (dereplication). These selected putative major capsid proteins were run in the MCP2T-RF model to predict capsid architectures.

## 3 Results

**Fig. 3:**
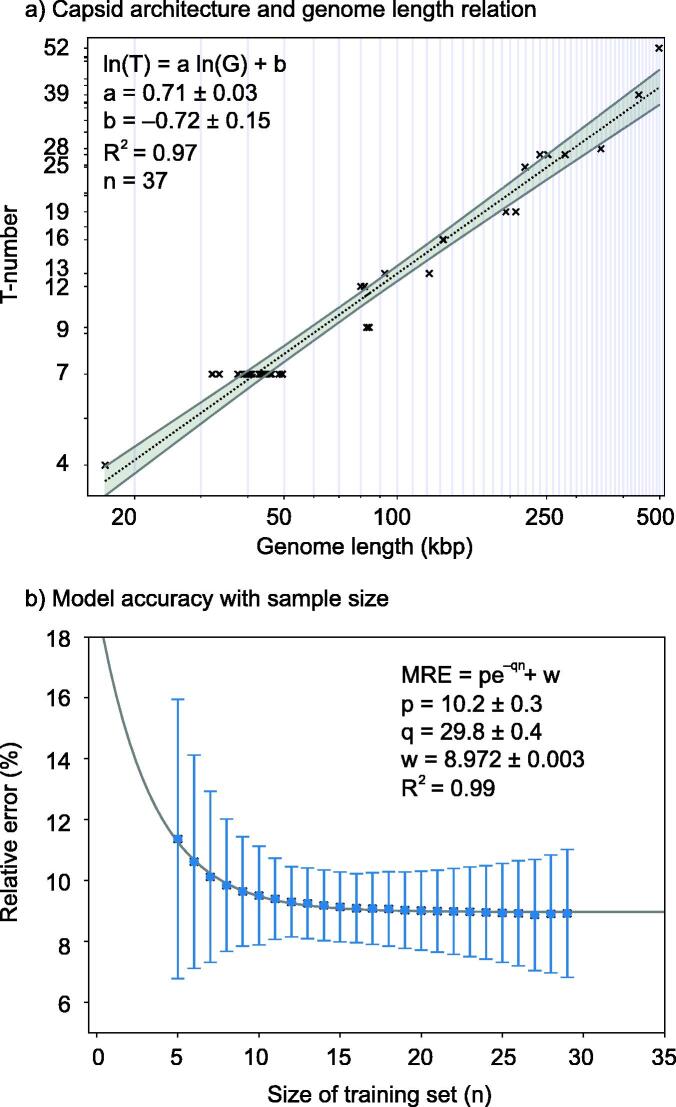
**Genome-to-T-number (G2T) model and accuracy.** a) T-number as a function of genome length in log–log scale (natural log) obtained from n = 37 tailed phage capsid 3D reconstructions (black product signs). The data is available in Data File 1. Vertical lines are displayed every 10 kbp as guide to the eye. The dotted black line corresponds to the linear regression of the power function (G2T) model in log–log scale (Eq. (2)). The gray band indicates the 95% confidence interval of the regression. b) Mean relative error, MRE, of the G2T model as a function of the size of the training set, n (blue squares). The error bars represent the standard deviation of the mean relative error. The solid, gray line corresponds to the fitted exponential decay model. a-b) The equations fitted, values of best fit, and coefficient of determination (R^2^) are displayed in each legend. (For interpretation of the references to colour in this figure legend, the reader is referred to the web version of this article.)

**Genome length predicts capsid architecture with 90% accuracy.** The power function model, Eq. (2), relating the capsid architecture, T, as a function of the genome length, G, explained 98% of the variance (R^2^ = 0.98, n = 37, Fig. 3a). This model is referred to as the genome-to-T-number (G2T) model. In the high-resolution capsid database, the genome lengths, G, ranged from G = 16.7 kilobase pairs (kbp) to 498.0 kbp. The capsid architectures ranged from T = 4 to 52 (see Data File 1). The fitted allometric exponent was 0.71 ± 0.03. This value was consistent with a prior analysis using a smaller dataset (0.68 ± 0.09, n = 23) [82]. The value was also close to the theoretical value, 2/3 ≈ 0.67 , expected for quasi-spherical shells packing a genome at a constant density (see supplementary section SI-2 for derivation). The mean relative error of the G2T model was 9% when testing the model using 30 structures for training and 7 for testing, 80/20 split. The analysis of the relative error using different training sizes revealed an initial exponential decay with training size, n, saturating at ∼ 9% for n ≥ 25 (R2 = 0.99, Fig. 3b). This implied that the genome length can predict the capsid architecture with 91% accuracy, each T-number is associated with a range of genome lengths that may overlap with nearby T-numbers (Data File 3), and this accuracy is not expected to improve when increasing the number of high-resolution capsid architectures.

**Fig. 4:**
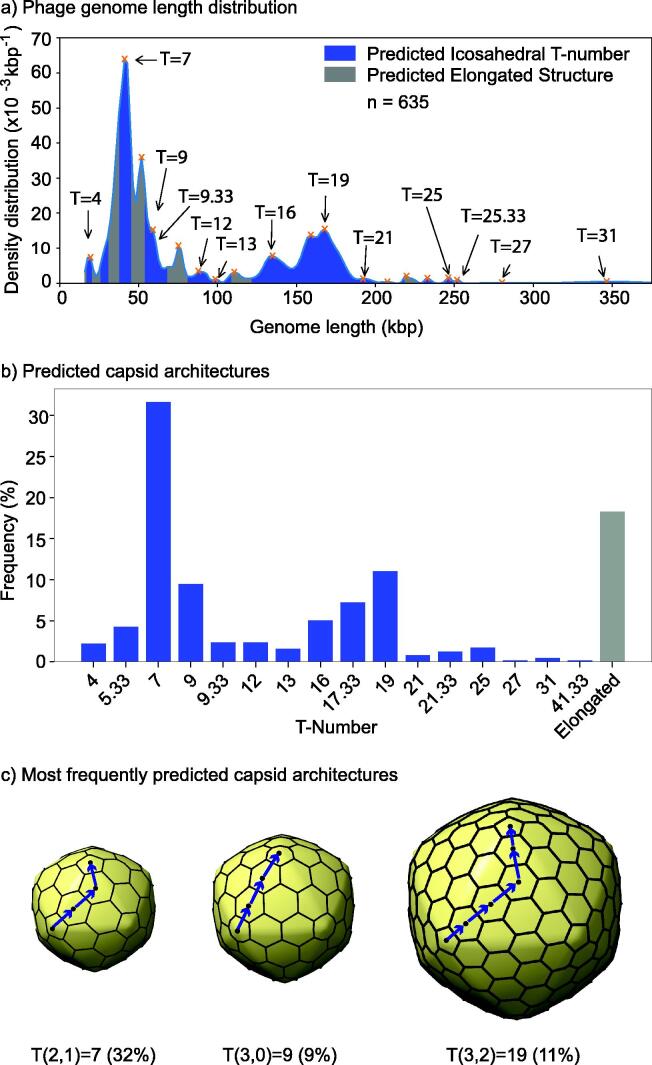
**Putative capsid architectures among phage isolates in the MCP library**. a) Probability density distribution of genome lengths (black line). The density was built with Gaussian kernels using multiple bandwidths (see methods). The genome length peaks in the probability density function are indicated with red product signs. Genome length regions predicted to form icosahedral capsids (G2T model) are shaded in blue. Regions associated with putative elongated capsids are shaded in gray. The T-numbers associated with peaks are displayed. b) Frequency of predicted architectures. The bar colors are associated with the shaded regions in panel a). c) 3D models for the three most common predicted capsid architectures. The labels at the bottom display the T-number, *h* and *k* steps, and frequency in percentage. Blue arrows and black dots highlight the steps in the hexagonal lattice. The models were generated with the *hkcage* function in Chimera X [96], [82]. (For interpretation of the references to colour in this figure legend, the reader is referred to the web version of this article.)

**Phage isolates display multimodal genome lengths dominated by T = 7, 9, and 19 architectures.** The genome length distribution of tailed phage genomes (n_lib_ = 635) displayed a multimodal distribution with 19 peaks (Fig. 4a). The densest genome regions were around ∼ 40 kbp and ∼ 160 kbp. The G2T model revealed that 15 out of the 19 peaks (55%) were associated with T-number architectures. Several possible T-number ranges overlap, thus yielding more than one possible T-number assignment for 38 % of phages (Supplementary Fig. S3). The remaining four peaks (21 %) were associated with alternative capsid architectures, which were interpreted as elongated architectures. The peak densities of elongated architectures, however, were far less prominent than those associated with icosahedral architectures. The total fraction of elongated architectures among isolates was predicted to be 18 % (Fig. 4b). This number was consistent with the observation of 10% to 20% of elongated architectures among isolates imaged with transmission electron microscopy [3]. Among the remaining 82 % of capsid architectures, which were predicted to be icosahedral, the most frequent capsids were T = 7 (32%), T = 9 (9 %), and T = 19 (11 %) (Fig. 4c). These three architectures combined accounted for 51 % of the putative structures. In the high-resolution database (Data File 1) 20 capsids were T = 7 (54%), no capsids were T = 9 (0%) and two capsids were T = 19 (5%). Therefore, with respect to tailed phage isolates, T = 7 has been over sampled in high-resolution capsid studies, while T = 9 and T = 19 have been under sampled. No tailed phage capsids were predicted to adopt the following T-numbers: 1, 1.33, 3, 25.33, 28, 33.33, 36, 37, 37.33, and 39.

**Fig. 5:**
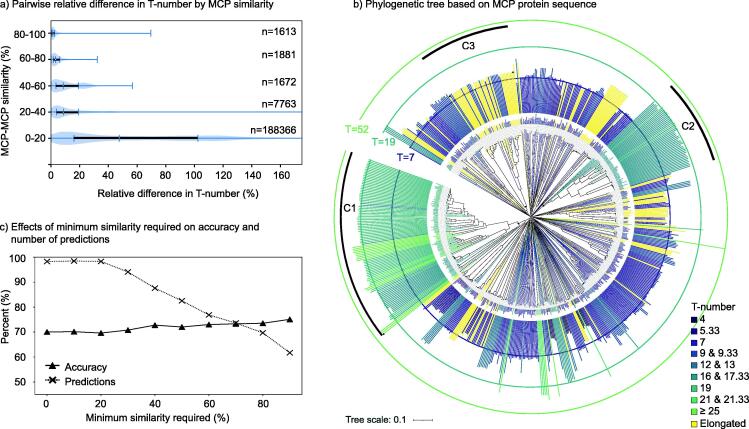
**Association between major capsid protein similarity and capsid architecture.** a) Violin plots for the distribution of relative differences in T-number (blue shade) for major capsid protein groups based on protein–protein similarity. The horizontal black line include ticks associated with the 25th quantile, median, and 75th quantile The blue lines capture the full range for each group. b) Unrooted circular phylogenetic tree obtained for the MCPs in the MCP/T library. The inner circle contains the phage names. The bars correspond to the genome length of the associated phage. The colors correspond to the predicted T-numbers and the three circumferences represent the mean genome length associated to the three more frequent T-number architectures. Three large clades are highlighted with black arcs and solid dots on the clade node. Data File 6 contains the tree in vectorial format. c) The percentages of total capsid architectures predicted (product signs) and accurate predictions (black triangles) are plotted as a function of the minimum protein–protein similarity required. The lines connecting points provide a guide to the eye. (For interpretation of the references to colour in this figure legend, the reader is referred to the web version of this article.)

**Protein sequence similarity can predict capsid architecture with 75% accuracy when requiring protein**–**protein similarities above 80%.** The analysis of the MCP/T library curated from phage isolates (n_lib_ = 635) revealed that MCPs sharing more than 80% similarity were associated with similar T-number architectures, with a mean relative difference in T-number of 2% (Fig. 5a). The relative T-number difference ranged from 0% to 7% for these highly similar MCPs. As the MCP similarity dropped below 60% the range of associated architectures increased substantially (Fig. 5a and Supplementary Table S1). In the last group, MCP similarities below 20%, the mean relative difference in T-number was 63 % with a broad range ranging from 0% to 699 %. A subset of 14.6 % of the MCPs that shared less than 20% similarity were predicted to form the same capsid architecture. This implies that high protein sequence similarity is a good predictor of capsid architecture, but very distant protein sequences can form the same capsid architecture.

The phylogenetic analysis of the major capsid protein sequences confirmed the observation derived from the initial MCP-MCP similarity analysis. The tree was very divergent due to the overall dissimilarity between proteins (Fig. 5b). Protein clusters displayed similar predicted architectures, but such architecture was not unique and could be find in independent clusters. Three clades contained a larger number of similar proteins, displaying each similar phage genome lengths and capsid architectures (see highlighted groups in Fig. 5c and Data File 5). Clade one (n*_c1_* = 95) adopted T = 19 capsids or slightly larger; clade two (n*_c2_* = 47) adopted T = 16 and T = 17.33 capsids, and clade three (n*_c3_* = 64) adopted mostly elongated architectures and some T = 9 and T = 9.33 architectures (all with similar genome lengths). These architectures were not exclusive of these clades; other small divergent clusters also adopt them. The frequent T = 7 capsids were distributed in multiple groups across the tree. The phylogenetic tree suggested that similar capsid architectures have emerged independently several times during tailed phage evolution. The alignment, Newick format tree, vectorial render of the tree, and nodes associated to the three highlighted clades are provided in Data Files 4–7.

The prediction of capsid architectures based on MCP-MCP similarity (MCP2T-PM model) assigned T-numbers to 98 % of the test set with 70 % accuracy when the proximity did not require a minimum similarity threshold to make a prediction (Fig. 5c). As the similarity percentage required to make a prediction increased, the accuracy increased slightly, reaching 75 % when requiring 90% similarity. However, above similarity thresholds of 20%, the number of possible predictions decreased substantially, reaching 61 % of the test dataset when requiring 90% similarity (Fig. 5b).

**Fig. 6:**
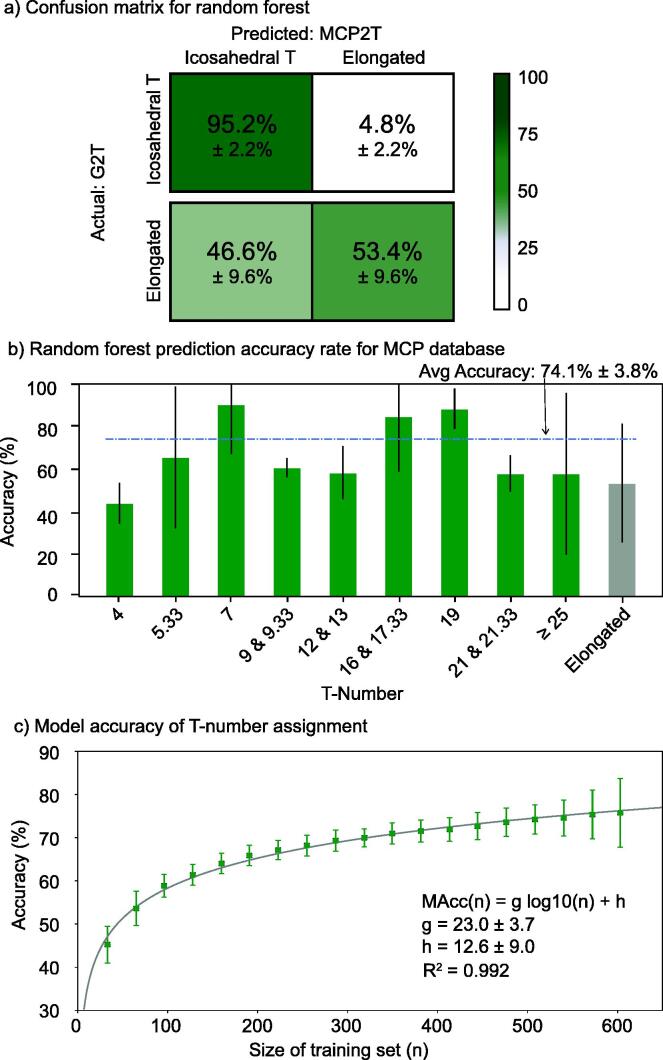
**Capsid architecture prediction from major capsid protein sequence composition.** a) Confusion matrix (mean and standard deviation) comparing actual capsid morphologies and predicted capsid morphologies for the major capsid protein-to-T-number random forest (MCP2T-RF) model. The green gradient scale reflects the mean values. b) Accuracy of the MCP2T-RF model predicting different architectures. Bars represent the mean accuracy (green for T-number architectures and gray for elongated architectures). Error bars display the standard deviation. The dashed line indicates the average accuracy. c) Mean (green squares) and standard deviation (error bars) accuracy of the MCP2T-RF model as a function of the size of the training set, n. The solid, gray line is the fitted logarithmic model displayed in the legend (equation, parameters, and coefficient of determination, R^2^). (For interpretation of the references to colour in this figure legend, the reader is referred to the web version of this article.)

**MCP amino-acid composition predicts capsid architecture with 74% accuracy.** The random forest model (MCP2T-RF) trained using the MCP/T library (n = 508 out of 635 in a 80/20 split) successfully identified on average 95.2% ± 2.2% of icosahedral structures as icosahedral, and 53.4% ± 9.6% of the elongated structures as elongated. (Fig. 6a). The accuracy varied across T-numbers (Fig. 6b). For T= , 7, 16–17.33, and 19, the accuracy was above 80%, while for T = 4 , the accuracy was just below 50%. The average accuracy was 74%. (see Supplementary Fig. S4 for further details on the T-number confusion matrix). The most relevant amino acid sequence features classifying the T-number were the amino acid length (len) and frequencies of glycine (G), alanine (A), and phenylalanine (F) (Supplementary Fig. S5).

To obtain further insight about the random forest model, the amino acid sequence features were investigated for the three large clades identified in the phylogenetic analysis. Clade 1 and clade 3 displayed, respectively, three and two amino acid sequence features in their MCPs that departed significantly from the average features in the MCP/T library (Supplementary Table S2). Clade 1 (characterized by T = 19 capsid structures) displayed MCP sequences with a larger average number of amino acids (498 versus 391), average glycine enrichment (9.6% of the sequence versus 7.8%), and average impoverishment of leucine (6.4% of the sequence versus 7.9%). Clade 3′s MCPs (characterized by elongated structures near T = 9 architectures) were on average enriched in tryptophan (1.5% of the sequence versus 0.9%) and impoverished in tyrosine (2.0% of the sequence versus 3.0%).

The accuracy of the model was investigated as a function of the size of the training data set. This identified a logarithmic increase of accuracy with the training size (R^2^ = 0.996, Fig. 6c). The accuracy model predicts that reaching a 90% accuracy would require a training set of 2,330 , that is, a library of 2,588 major capsids proteins and putative capsid architectures.

The training time of the random forest model increased linearly with the size of the training data set (slope = 2 ms/datum, R^2^ = 1.00, Supplementary Fig. S6a). Training the random forest model with a training set of size 2,330 (predicted to be 90% accurate) would take about 20 s. The increase in training time was about two times less costly than for the similarity model (slope = 4 ms/datum, R^2^ = 1.00, Fig. SI-7a). In the random forest model, a single prediction was independent of the training size, approximately 1 ms for a single search (Fig. SI-7b). For the similarity model, the search time was faster for small training sizes, but it increased quadratically with the training size, that is, *O(n^2^)* (Supplementary Fig. S6b). The crossover time-search was around training size sets of size 10,000, with a search time on the order of 1 ms. Therefore, the random forest model provided a scalable approach.

**Fig. 7:**
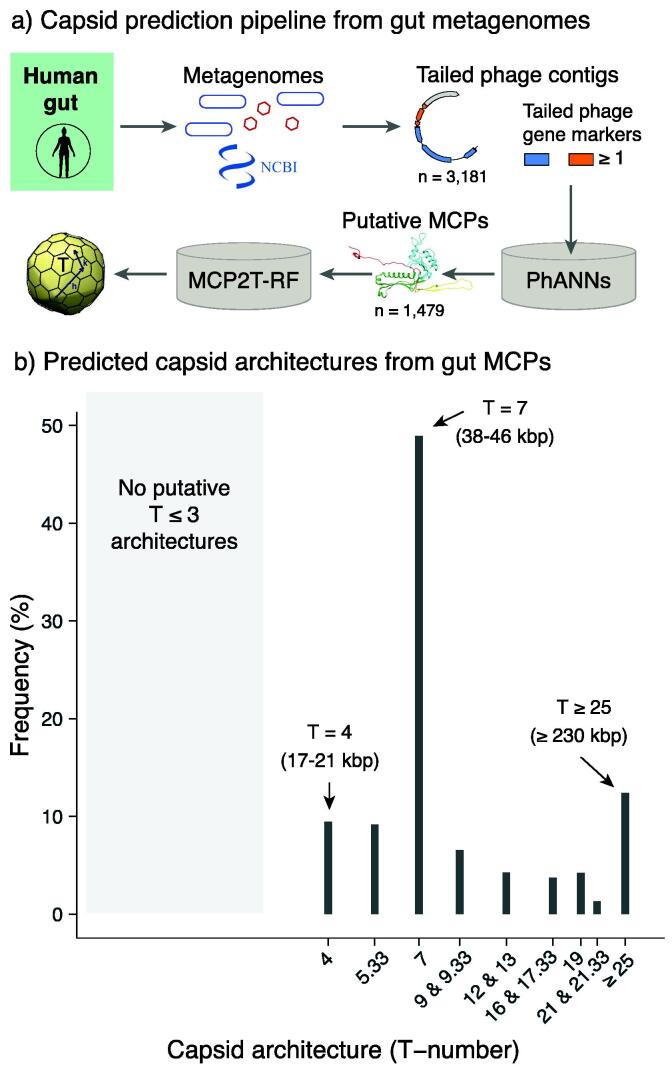
**Capsid architectures predicted in gut metagenomes.** a) Bioinformatic pipeline displaying the key steps and tools used to predict tailed phage capsids from gut metagenomic data. b) Frequency of predicted icosahedral capsid architectures. The arrows highlight the three most frequent T-numbers, including the putative genome length range in parenthesis.

**T = 7 capsids dominate among uncultured gut phages.** A total of distinct 1,479 HK97-fold major capsid proteins annotations were identified among 3,181 metagenomically assembled genomes from gut samples containing tailed phage markers and direct terminal repeats (Fig. 7a). The MCP2T-RF model predicted the presence of capsid architectures ranging from T = 4 to T = 31 (Data File 8). The most frequent predicted capsid architecture was T = 7 (48.9%), followed by T ≥ 25 (12.4%), T = 4 (9.4%), and T = 5.33 (9.2%) (Fig. 7b). The frequency of predicted elongated capsids was 1.9% (see Data File 9). The frequency of putative T = 7 capsid architectures in gut metagenomes was ten points larger than those predicted among tailed phage isolates (Fig. 4b and 7b). This was interpreted due to the large presence of integrated prophages in bacterial genomes in the gut[58], [83]. The genome length of phages that can integrate as prophages is typically around 45 kbp [14], which is within genome length that we predict to be associated with T = 7 capsids. The large frequency of T ≥ 25 architectures in gut metagenomes was unexpected based on tailed phage isolates, probably because the observation of jumbo phages has been particularly elusive until the emergence of sequencing [12].

## 4 Discussion

The computational model introduced here confirmed a strong association between the information encoded in the major capsid protein and the capsid architecture of tailed phages. The application of this model to metagenomic data facilitated surveying the putative capsid architectures of tailed phages in the human gut microbiome. The most frequent capsid predicted was T = 7. High-resolution studies have revealed that this architecture is common among tailed phages [118]. Our interpretation is that the high frequency of T = 7 capsids is associated with the prevalence of lysogeny in gut bacteria [109], [83]. Temperate tailed phages can integrate in bacterial genomes as prophages, forming lysogenic bacteria that can alter the functionality of microbiomes [68], [58]. These prophages are expected to be present in gut metagenomes in addition to free tailed phages. Temperate phages are characterized by adopting genomes around 45 kbp [14], which, based on our model, are expected to be associated with T = 7 capsids, as observed in lambda and other temperate lambdoids [23]. Prophages in bacteria can be domesticated and shortened in genome length [14], but the remaining major capsid protein would indicate that the free version of the prophage was encoding a T = 7 capsid.

The gut metagenome analysis also identified a significant presence of T ≥ 25 capsids with predicted genome lengths above 206 kbp (Fig. 7b). This was an unexpected result because these group of capsids are relatively rare among tailed phage isolates (Fig. 4). Nonetheless, these capsids are considered jumbo phages (above 200 kbp) [126], and recent studies have discovered that they are far more common than initially expected across ecosystems [45], [62], [12]. Our analysis indicates that jumbo tailed phages might be particularly prevalent in gut microbiomes, in agreement with recent studies [33] The detailed genomic and structural characterization of jumbo phages might be key to understanding the ecology of phage and bacteria in the human gut. Additionally, the model also predicted more frequent small capsids, T = 4 and T = 5.33 (Fig. 7b) than expected from phage isolates. This also aligns with recent bioinformatic studies indicating that these groups of capsids have been under sampled [82], [12]. These small capsids could be the key to understand the evolution of tailed phages and cellular compartments like encapsulins [82]. The application of the G2T model to the circular genome lengths indicated that 43 genomes predicted T = 4 and T = 5.33 capsids in agreement with the MCP2T-RF model; only 56 genomes led to this agreement for gut phage genomes. This result suggests that these 43 candidates are probably complete phage genomes that could provide a great source of information to investigate the structure and evolution of small, tailed phage capsids. The MCP2T-RF model did not predict smaller capsids (T less than 4) because such putative capsids were not present in the MCP/T library, but the observation of small circular genomes among tailed phages suggest that that they could exist [82].

The computational model introduced here is a first step to bridge viral genomic information with viral structural phenotype in microbiomes. However, there are important steps ahead to improve the accuracy of the models. The MCP2T-RF model is projected to reach an accuracy of 90% using a library of 2,600 MCPs and putative T-number architectures (Fig. 6c). However, to go beyond this accuracy, it would be necessary first to improve the underlying genome-to-T-number (G2T) model responsible for building the MCP/T library (Fig. 2). The G2T model currently has an accuracy of 91%, but this error is not projected to be reduced when increasing the number of structures in the high-resolution database (Fig. 3b). This implies that at least one more genome feature would be necessary in addition to the genome length. One compelling direction would be to add the tailed phage packing strategy. Head-full mechanisms tend to pack more DNA than encoded in the genome, while packing signal mechanisms pack exactly the genome length [22], [59]. These variations may explain that the empirical exponent in the power-function model is slightly larger than the theoretical prediction (Fig. 3a).

The research introduced here does not clarify the structural reasons why features such as amino acid sequence length as well as glycine and threonine frequencies are so relevant in predicting capsid architecture. Nonetheless, two out of the three big MCP clades identified phylogenetically displayed a few characteristic features, including larger amino acid sequence lengths and a larger content of glycine and tryptophan, which could be important to MCPs associated with large capsids. However, follow-up structural analyses would be necessary to reveal the origin of the selection of MCPs to form specific T-numbers. Additionally, information from other proteins involved in the assembly of tailed phages (like scaffold, minor capsid proteins, and reinforcement proteins) will be necessary to predict more accurately the capsid architecture as well as alternative capsid architectures formed by the same major capsid protein [73], [44], [32], [99]. It is now possible to predict these protein functions from genomic data, but the accuracy is typically lower than for major capsid proteins, and some categories are still hard to predict correctly, like minor capsid proteins [21].

The method described in Fig. 2 could be adapted to also predict the capsid architecture of other viruses. The first key step would be identifying strong allometric relationships between the genome length and capsid architecture of those viruses (Fig. 2a). The analysis of allometric relationship between virion volume and genome length combining all virus types has led to non-optimal statistical results due to the variance between virus groups [30], [15], [37]. Prior studies indicate that the allometric exponent would vary strongly depending on the virus group [10], [37]. A strategy to improve the accuracy of this relationship is separating viruses that use the same capsid protein fold and genome storage strategy[1], [70], [122], [69], [101]. The second step would be generating the MCP/T library of capsid proteins and capsid architectures using isolated genomes (Fig. 2b), and the third would be using these libraries to train similar statistical learning methods as those presented here (Fig. 2c). Sequencing technologies are now capable of identifying both DNA and RNA viruses [104], [43]. Nonetheless, the diversity of capsid architectures among viruses different than tailed phages is smaller [89]. Thus, other phenotypical features might be more interesting to include in the MCP/T library. The development of bioinformatic pipelines as the one used here would facilitate constant monitoring and analysis of viral capsids of different virus groups in the environment (Fig. 7a).

## 5 Conclusion

The protein-to-capsid model introduced here predicts the architecture of tailed phages from just one gene (the major capsid protein) with 74% accuracy. Increasing the library of proteins and putative architectures around 2,600 could increase this accuracy to 90%. The application of this approach in human gut metagenomes predicted the abundance of T = 7 capsids probably associated to temperate phages followed by an unexpected abundance of jumbo capsid architectures (T ≥ 25) and small architectures (T = 4 and T = 5.33) that have been under sampled among phage isolates and high-resolution tailed phage capsid studies. The method introduced here will facilitate bridging the evolution and selection of tailed phage genomic data with capsid architecture. This would eventually help identify the functions associated with capsids beyond storage capacity.

## Funding

The research of D.Y.L., C. B., and A.L. was supported by the National Science Foundation award #1951678 and the Gordon and Betty moore Foundation, GBMF9871, grant https://doi.org/10.37807/GBMF9871 and D.Y.L.'s research was also supported by the a STEM scholarship award funded by the National Science Foundation grant DUE-1259951. The research of M.A.S. was supported by the National Institutes of Health GM110588 and the California Metabolic Research Foundation.

## CRediT authorship contribution statement

**Diana Y. Lee:** Conceptualization, Data curation, Methodology, Writing – original draft. **Caitlin Bartels:** Data curation. **Katelyn McNair:** Data curation. **Robert A. Edwards:** Data curation. **Manal A. Swairjo:** Data curation, Writing – review & editing. **Antoni Luque:** Conceptualization, Methodology, Writing – review & editing.

## Declaration of Competing Interest

The authors declare that they have no known competing financial interests or personal relationships that could have appeared to influence the work reported in this paper.

## Figures and Tables

**Supplementary figure 1:**