# Supplementary figures and images for: Predicting the capsid architecture of phages from metagenomic data
Source: Comput Struct Biotechnol J. 2022 Jan 5;20:721–32. doi: 10.1016/j.csbj.2021.12.032 (PMC8814770; doi:10.1016/j.csbj.2021.12.032)

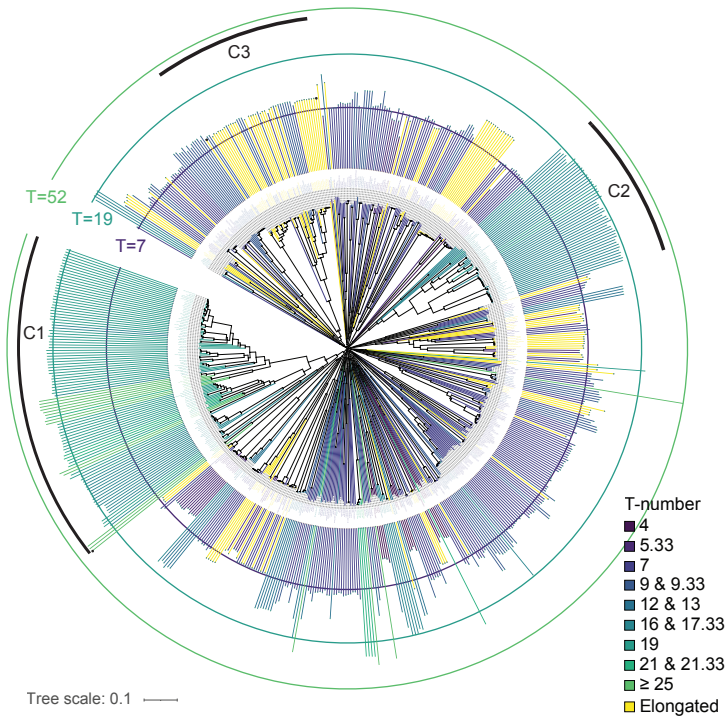

Supplement: Supplementary data 6 [file mmc6.pdf]
