# Supplementary material for: Predicting the capsid architecture of phages from metagenomic data
Source: Comput Struct Biotechnol J. 2022 Jan 5;20:721–32. doi: 10.1016/j.csbj.2021.12.032 (PMC8814770; doi:10.1016/j.csbj.2021.12.032)
Supplement: Supplementary data 10 [file mmc10.pdf]

SUPPLEMENTARY INFORMATION

Predicting the capsid architecture of phages from metagenomic data

Diana Y. Lee[1,2], Caitlin Bartels[1,3], Katelyn McNair[1,2], Robert A. Edwards[1,2,3,4], Manal A. Swairjo[1,5], and Antoni Luque[1,2,6,*]

[1] Viral Information Institute, San Diego State University, 5500 Campanile Drive, San Diego, CA, 92182, USA.
[2] Computational Science Research Center, San Diego State University, 5500 Campanile Drive, San Diego, CA, 92182, USA.
[3] Department of Biology, San Diego State University, 5500 Campanile Drive, San Diego, CA, 92182, USA.
[4] Flinders Accelerator for Microbiome Exploration, Flinders University, Bedford Park, GPO Box 2100, Adelaide 5001, South Australia, Australia.
[5] Department of Chemistry and Biochemistry, San Diego State University, 5500 Campanile Drive, San Diego, CA, 92182, USA.
[6] Department of Mathematics & Statistics, San Diego State University, 5500 Campanile Drive, San Diego, CA, 92182, USA.
[*] Corresponding author: aluque@sdsu.edu

SI-1. Description of Data Files

Data File 1 (data_file_1_high_resolution_phages.csv): This file contains the high-resolution phage data that was used to create the statistical model. The columns of the data file are: *Capsid:* Name of the structure, *T:* T-Number, *Lattice*: the lattice identified as hexagonal (“hex”) or tri- hexagonal (“tri-hex”), *h*: the h value for the lattice, *k*: the k value for the lattice, *Max Diameter (nm)*: maximum diameter of the capsid. *Family*: phage family Siphoviridae (“S”), Myoviridae (“M”), or Podoviridae (“P”), *Reference*: the references for the data, *DNA (bp)*: Genome length in basepairs, *DNA (kbp)*: Genome length in kilobasepairs, *PDB/ EMDB:* the Protein Data Bank (PDB) or Electron Microscopy Data Bank (EMDB) reference for the high-resolution microscopy.

Data File 2 (data_file_2_MCP_Genome_database.csv): This file contains the MCP Genome data that was used to create the MCP-T library. The columns of the data file are: *ID:* Virus ID given to the structure for this project, *DEFINITION:* Name of the structure, *HR data:* If the structure is on the high resolution phages, this column contains an “X”, *HR_T:* If the structure is on the high resolution phages, this column contains the T-number as identified in the HR phage data file, *COMPLETE_GENOME:* NCBI nucleotide sequence reference for the phage, *COMPLETE_GENOME_BP:* Genome length of the phage in basepairs, *NCBI_GENPEPT_PROTEIN_ID:* NCBI reference for the protein ID for the Major Capsid Protein (MCP) gene, *PROTEIN_PRODUCT:* NCBI gene description, *PROTEIN_BP:* Length of the MCP in basepairs, *TRANSLATION:* MCP protein sequence.

Data File 3 (Data_File_3_T-number_and_genome_length_ranges.csv): This file contains the T-numbers and the genome length ranges associated with each T-number from 1 through 52. The columns of the data file are: *T-number:* Each possible T-number from T=1 through T=52, *lower_end_kbp*: the lower end of the kbp range for genome length for each T-number (mean - 9%), *mean_kbp*: the kbp for genome length at each T-number, *upper_end_kbp*: the lower end of the kbp range for genome length for each T-number (mean + 9%).

Data File 4 (data_file_4_MCPdb_clustal_omega_Align.txt): Multiple sequence alignment file for the MCP genome database as created using Clustal Omega (1.2.4).

Data File 5 (data_file_5_MCPdb_clustal_omega_Tree.txt): Newick tree file for the MCP genome database as created using Clustal Omega (1.2.4).

Data File 6 (data_file_6_tree_vectorial_format.pdf): Vectorial pdf format of the tree included in Figure 5b.

Data File 7 (data_file_7_clade_nodes.csv): This file contains the IDs of the three clades that were identified for analysis. The columns of the data file are: *clade 1 (19) id:* The IDs of the phages that are included in clade 1*, clade 1 (19) description*: The names of the phages that are included in clade 1, *clade 2 (16 & 17.33) id:* The IDs of the phages that are included in clade 2*, clade 2 (16 & 17.33) description*: The names of the phages that are included in clade 2, *clade 3 (9/9.33/e) id*: The IDs of the phages that are included in clade 3, *clade 3 (9/9.33/e) description:* The names of the phages that are included in clade 3.

Data File 8 (data_file_8_gut_phage_data.csv): This file contains the gut data to which we applied the MCP2T-RF model. The columns of the data file are: *Organism:* NCBI nucleotide sequence reference for the phage, *ORF_ID,* ORF ID of the MCP gene, *Max_PHANNs_Score:* Maximum score from the PHANNs webserver results, *Max_Score_Category:* Maximum score catergory from the PHANNs webserver results, *Genome_Len:* Genome length of the phage in basepairs, *MCP_Seq:* MCP protein sequence, *MCP_Len:* Length of the MCP in basepairs, *IsoElectric Pt:* Isoelectric point of the MCP sequence as calculated by biopython, *Predicted_T(RF)*: The T-number as predicted by the MCP2T model.

Data File 9 (data_file_9_predicted_capsids_gut_metagen.txt): This file contains the frequency of T-numbers predicted for the gut data sorted by highest frequency of prediction. The columns of the data file

are: *T-num*: The T-number as predicted by the MCP2T model, *Frequency:* number of phages predicted at this T-number, *Percent*: Percent of total phages predicted at this T-number, *Cum. Percent:* cumulative percent of phages represented.

SI-2. Theoretical derivation of the model. To build the model relating DNA volume to T-number, we begin with the formula for the volume of a regular icosahedron based on the edge length, a. In this derivation, all lengths are measured in nanometers (nm):

$$V = \frac{5}{12}a^3\left(3 + 2\sqrt{5}\right) \tag{1}$$

Where the circumsphere radius *r* (nm) relates to the edge length *a* via the following relation:

$$a = \frac{4r}{\left(10 + 2\sqrt{5}\right)} \tag{2}$$

We can combine equations (1) and (2) to arrive at:

$$V = \frac{5}{12}\left(3 + 2\sqrt{5}\right)\left(\frac{4r}{\left(10 + 2\sqrt{5}\right)}\right)^3 = \frac{80\left(3 + 2\sqrt{5}\right)}{3\left(10 + 2\sqrt{5}\right)^3}r^3 \tag{3}$$

We can similarly write the surface area *A* as:

$$A = 5\sqrt{3}\left(\frac{4r}{\left(10 + 2\sqrt{5}\right)}\right)^2 \tag{4}$$

And leave this equation for a moment to consider the T-number. Phage capsids were traditionally considered to be built from groups of proteins, or capsomers-- specifically, groups of six proteins (hexamers). For an icosahedron to be built from hexagons, 12 hexagons must become pentagons to be folded into the corners, thus in folding a phage capsid, 12 hexamers become groups of five proteins, or pentamers. The formula for the T-number then is:

$$T = h^2 + hk + k^2 \tag{5}$$

Where h and k are integers describing the steps in numbers of proteins from one pentamer to another. The total number of proteins in an icosahedral capsid is then 60T. Because there are always 12 pentamers, the number of hexamers must be 10(T-1), and the total number of capsomers on a shell can be described as:

$$N = 12 + 10(T-1) = 10T + 2 \quad (6)$$

We now assign the radius of one of those capsomers to be σ_0. The surface area of each hexamer, A_H can be described as:

$$A_H = 2\sqrt{3}\sigma_0^2 \quad (7)$$

And the surface area of each pentamer, A_P can be described as:

$$A_P = \frac{5}{6}A_H \quad (8)$$

By rearranging the formula for *A* (eq. 4) above, we get:

$$r = \sqrt{A\left(\frac{5+\sqrt{5}}{40\sqrt{3}}\right)} \quad (9)$$

We know that surface area A is equivalent to the sum of the total surface areas of the hexamers and pentamers, or:

$$A = 10(T-1)A_H + 12A_P \quad (10)$$

We can then replace the area in the radius formula in terms of σ_0:

$$r = \sqrt{\left(\frac{5+\sqrt{5}}{40\sqrt{3}}\right)\left(20\sqrt{3}(T-1) + \frac{5\sqrt{3}}{3}\right)\sigma_0^2} \quad (11)$$

Which can be simplified to:

$$r = \sqrt{\left(\frac{5+\sqrt{5}}{2}\right) T\, \sigma_0} \tag{12}$$

(Luque thesis). To relate the volume of DNA in kilobasepairs (kbp) to the volume of the icosahedron (nm^3), we need to address the density of DNA, ρ (kbp/nm^3):

$$V = DNA * \rho \tag{13}$$

Using the formula for the volume of an icosahedron in terms of its circumsphere radius above (eq. 3), and the radius in terms of T and σ_0 (eq. 12):

$$DNA * \rho = \frac{80\left(3+2\sqrt{5}\right)}{3\left(10+2\sqrt{5}\right)^{3}} \left(\sqrt{\left(\frac{5+\sqrt{5}}{2}\right) T}\right)^{3} \sigma_0^3 \tag{14}$$

From here, we can finally solve for T in terms of DNA:

$$T = DNA^{\frac{2}{3}} * \rho^{\frac{2}{3}} \left(\frac{3}{80\left(3+2\sqrt{5}\right)}\right)^{\frac{2}{3}} \left(\frac{20+4\sqrt{5}}{5+\sqrt{5}}\right) \frac{1}{\sigma_0^2} \tag{15}$$

At this point, we assume σ_0 and ρ to be constant (Luque et al. 2020), thus equation 15 can be expressed as:

$$T = c_1 DNA^{\frac{2}{3}} \tag{16}$$

Where c_1 is some constant.

SI-3. Statistical linear regression. We examined the correlation of the DNA size in kilobase pairs versus the T number and built a linear regression directly from the raw data. The regression yielded an R^2 correlation coefficient of 0.974. When the residuals were plotted against the independent variable and examined however, the resulting plot appeared to exhibit a pattern. Various other methods of data transformation were then tested to arrive at the method that yielded the highest R^2, the power transform:

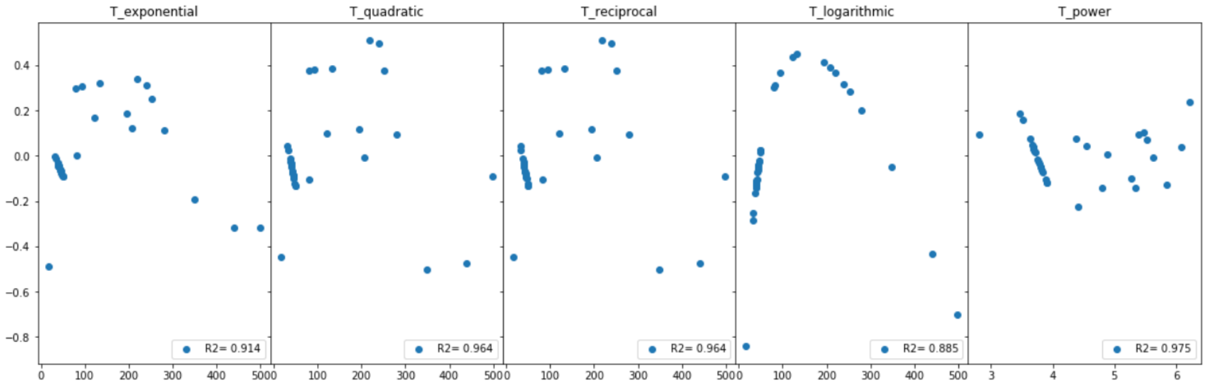

Figure S1. Residuals for the different genome-to-models tested.

As another option, robust least-squares optimization on a function of the form:

$$T = a * DNA^b$$

With adjustments to the parameters to utilize loss type 'soft_l1' to minimize the effects of outliers gave us a non-linear regression that yielded at best, an R^2 of 0.949, making it clear that the linear regression on the power transformed data is still the best estimator for this dataset.

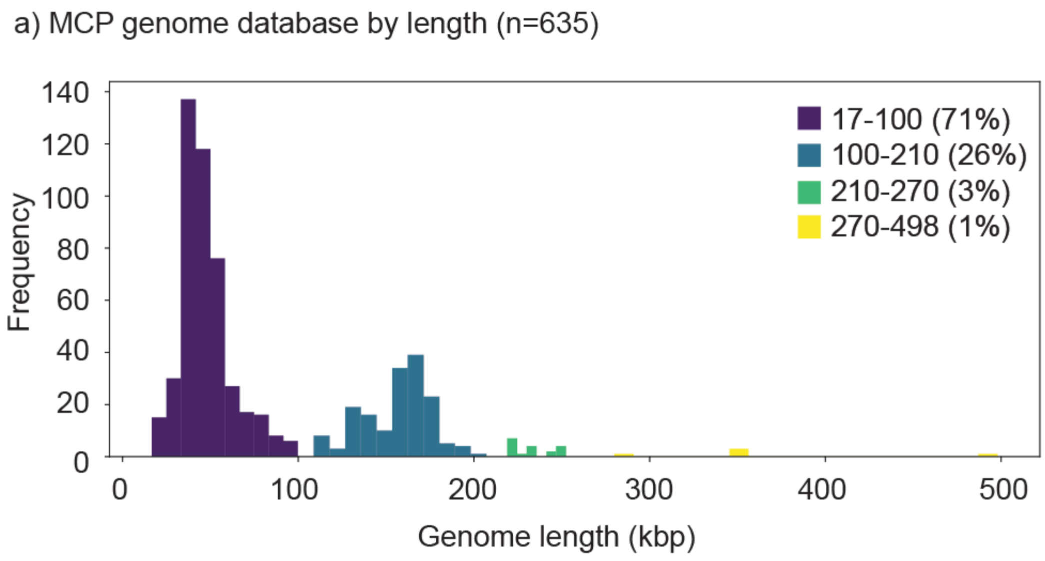

Figure S2. **MCP genome database histogram.** a) The MCP genome database (n=635) visualized as a histogram shows four distinct groups of genome lengths: 17-100 kbp, which represents n= 450 or 71% of the database, 100-210 kbp, representing n=162 or 26% of the database, 210-270 kbp, representing 18 structures or 3% of the database, and 270-498 kbp, representing n=5, or <1% of the database.

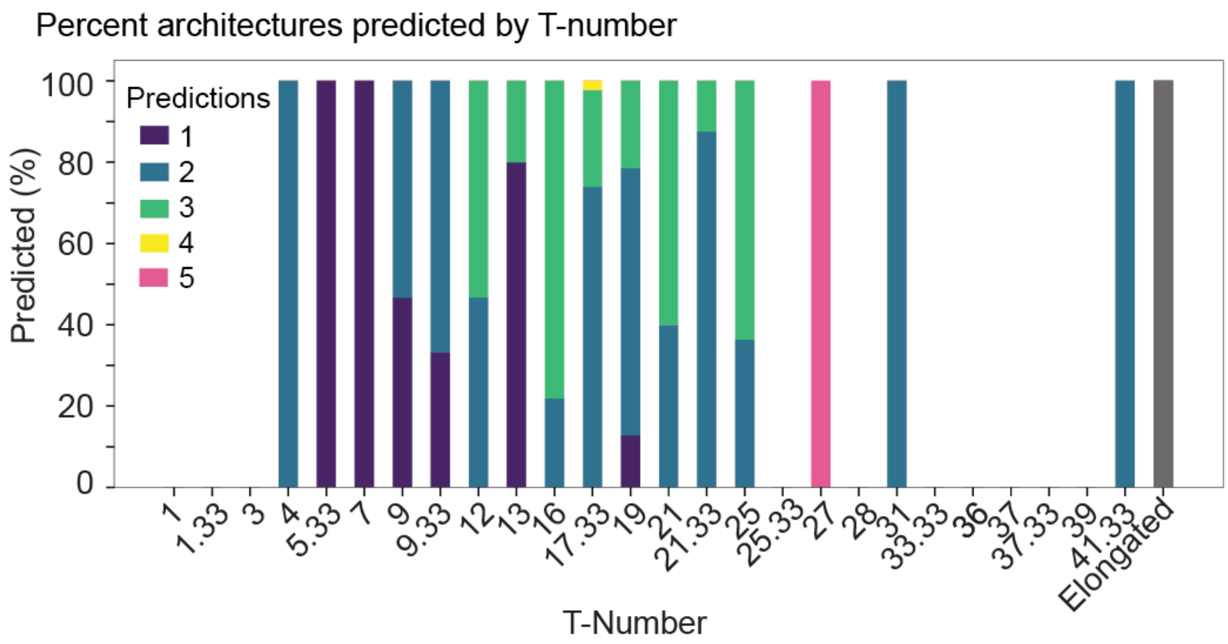

Figure S3. Due to the margin of error of 9%, there were some structures whose calculated T-number based on the G2T model that fell into ranges of more than one possible T-number. While we assigned the nearest T-number to these structures, it is instructive to see where the areas of highest overlap occurred. Here, for each T-number, the percent of structures that were assigned to 1, 2, 3, 4 or 5 possible T-numbers are visualized as percentages for each T-number.

Table S1. **Pairwise similarity table.** Phages in the MCP database (n=635) categorized by pairwise similarity and characterized by relative difference in predicted T-number.

Pairwise similarity (%)	Relative difference in predicted T-number (%)			
	minimum	maximum	mean	median
[0-20)	0	699	63	48
[20-40)	0	197	15	9
[40-60)	0	57	11	8
[60-80)	0	32	6	3
[80-100]	0	7	2	1

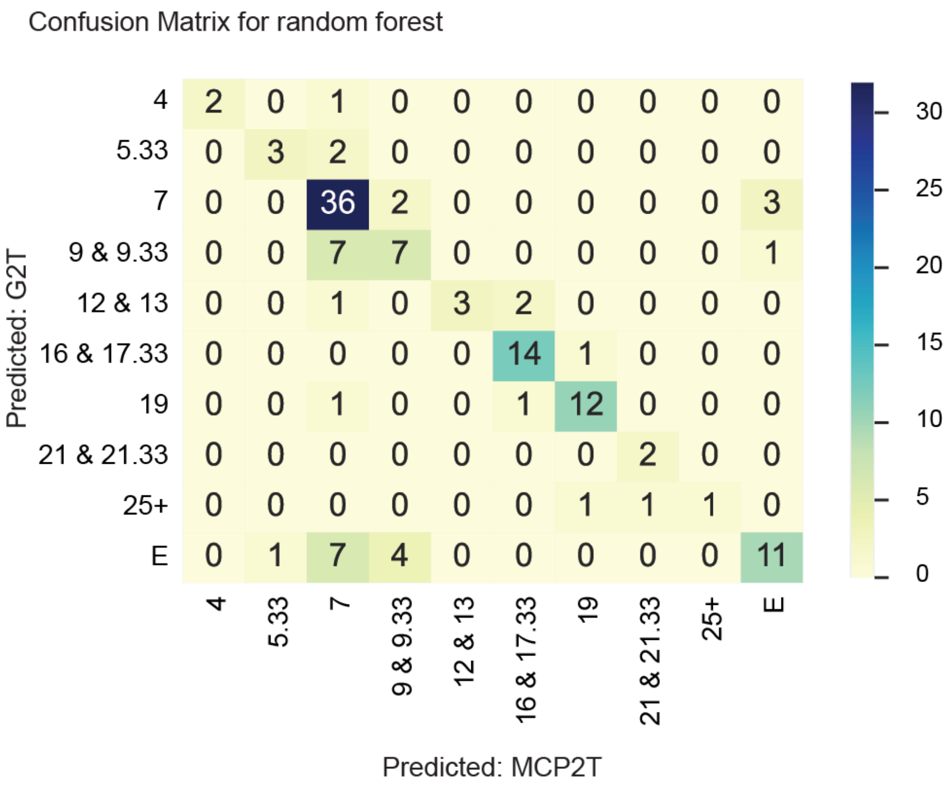

Figure S4. Multi-class confusion Matrix for Random Forest. a) A confusion matrix for the random forest trained on the available MCP data shows the T-numbers as predicted by the G2T model as the rows, and the T-numbers as predicted by the random forest as the columns. The intersection of the rows and columns on the diagonal represent the structures that were predicted correctly, in this case 91 of the 123 structures, or 74%. Here 'E ' indicates elongated structures.

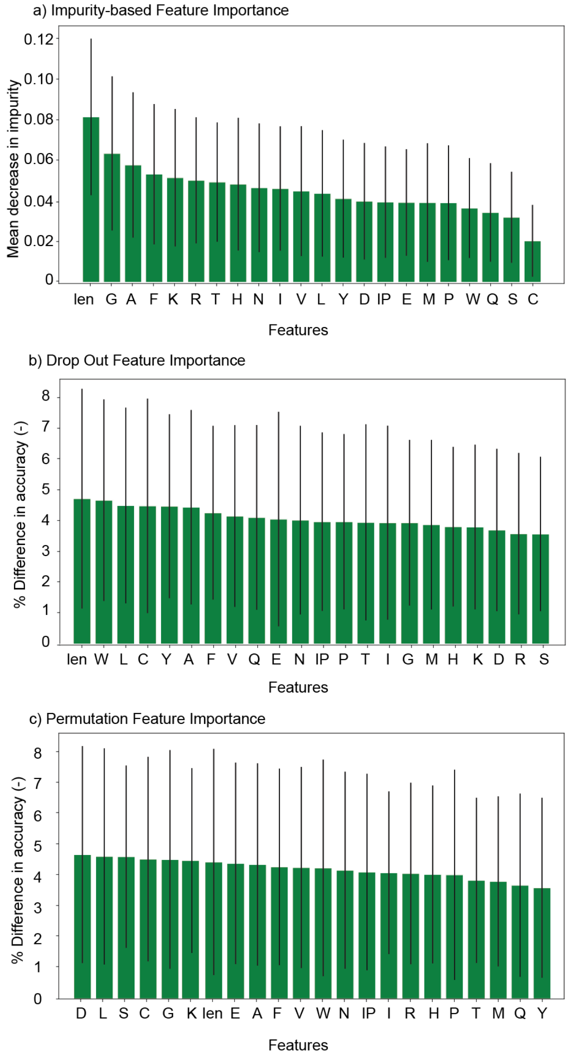

Figure **S5. Random forest impurity, dropout, and permutation analyses. Impurity-based feature importance endemic to Sci-kit Learn (Pedregosa et. al 2011) as well as** dropout and permutation analyses were used to assess the predictive importance of every single individual feature. In the impurity-based feature importance (a), Sci-kit Learn calculates the mean decrease in impurity for each feature. In the dropout analysis (b), one feature was removed from the dataset, the random forest was retrained, and the accuracy re-examined. For the permutation analysis (c), the values of a single feature were randomized across the dataset, the random forest was retrained, and the accuracy re-examined.

Comparing the new accuracy to the baseline accuracy yielded a percentage difference that represents the importance of the feature in question. This procedure was repeated for each of the 22 features in use, 1000 times each for statistical robustness. The features used in the dataset are on the x axis, where ‘len’ is length of the MCP sequence, ‘IP’ is the isoelectric point, and all other features are the relative frequency of each corresponding amino acid in the MCP sequence.

Table S2. Comparison of protein sequence features for the MCP/T library and the MCP clades C1, C2, and C3. The columns include the mean, standard deviation, and relative increase of the clades with respect the full library. The values highlighted in yellow were considered significant (more than a standard deviation difference).

Feature	all mean	std dev	C1 mean	C1 ±	C2 mean	C2 ±	C3 mean	C3 ±
**IP**	5.248	0.515	5.200	-0.9%	5.277	0.5%	4.817	-8.2%
**len**	391.082	82.037	497.526	27.2%	465.085	18.9%	318.344	-18.6%
**A**	0.108	0.022	0.126	16.6%	0.095	-12.0%	0.116	7.5%
**R**	0.043	0.013	0.042	-3.5%	0.037	-14.9%	0.036	-15.6%
**N**	0.049	0.012	0.048	-2.5%	0.056	14.2%	0.044	-10.0%
**D**	0.065	0.012	0.055	-16.5%	0.062	-4.7%	0.069	5.3%
**C**	0.002	0.003	0.003	48.4%	0.000	-95.6%	0.001	-51.2%
**Q**	0.043	0.011	0.048	11.0%	0.053	23.2%	0.041	-5.7%
**E**	0.060	0.015	0.058	-3.1%	0.057	-3.8%	0.050	-16.9%
**G**	0.078	0.018	0.096	22.7%	0.064	-18.5%	0.089	13.6%
**H**	0.011	0.005	0.009	-20.6%	0.014	27.5%	0.013	20.8%
**I**	0.057	0.011	0.061	8.7%	0.060	5.4%	0.053	-5.6%
**L**	0.079	0.014	0.064	-19.0%	0.079	-0.8%	0.081	2.5%
**K**	0.057	0.019	0.050	-12.6%	0.060	4.8%	0.043	-24.5%
**M**	0.024	0.009	0.029	21.5%	0.026	8.1%	0.018	-26.9%
**F**	0.039	0.009	0.042	7.5%	0.039	0.1%	0.039	-1.9%
**P**	0.040	0.010	0.041	1.6%	0.041	3.3%	0.048	18.7%
**S**	0.057	0.011	0.057	-0.3%	0.061	7.9%	0.054	-4.6%
**T**	0.071	0.014	0.061	-13.1%	0.070	-1.0%	0.082	16.1%
**W**	0.009	0.005	0.008	-14.4%	0.008	-15.8%	0.015	56.1%
**Y**	0.030	0.009	0.036	18.9%	0.030	0.2%	0.020	-33.0%
**V**	0.077	0.014	0.067	-13.1%	0.087	13.5%	0.088	14.9%

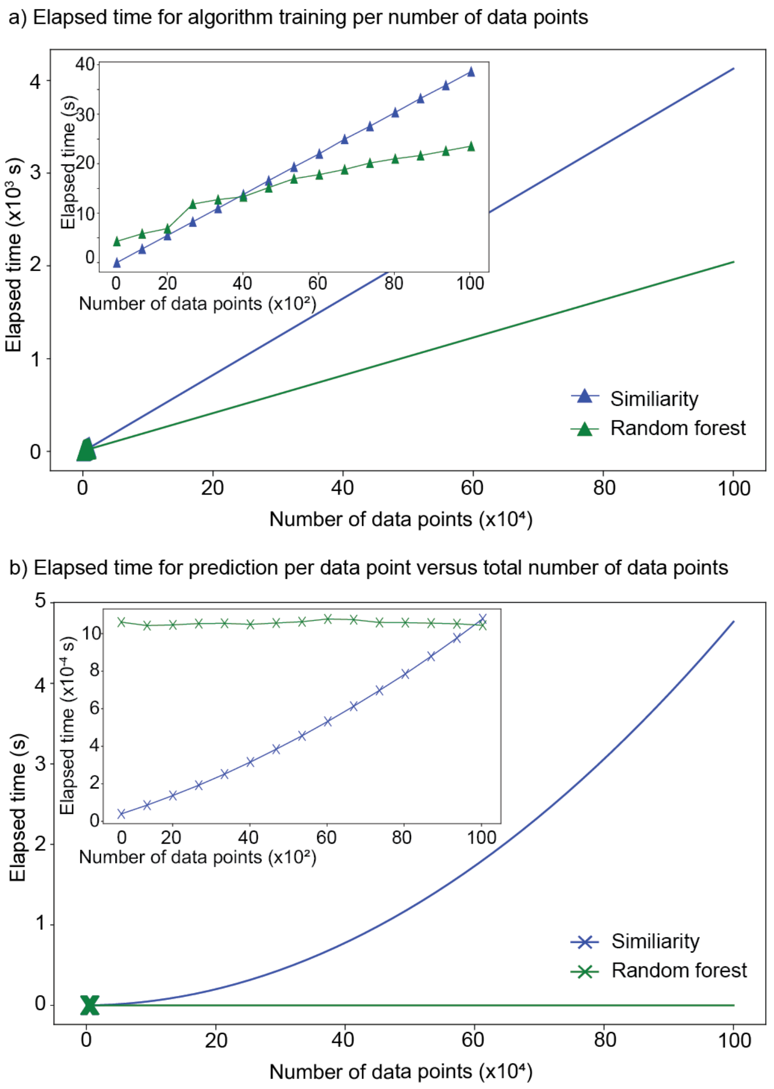

Figure S6. **Algorithm performance of MCP similarity versus random forest.** a) The inset shows the elapsed time in seconds required for training each of the MCP similarity (blue lines and triangles) and random forest (MCP2T)(green lines and triangles) algorithms as measured by test data to 10K data points. The larger figure shows the extrapolation of those linear trends out to a hypothetical 1M data points. b) The inset shows the elapsed time in seconds required for prediction of a single data point for each of the MCP similarity (blue lines and product signs) and random forest (MCP2T)(green lines and product signs) algorithms as measured by test data to 10K data points. The larger figure shows the extrapolation of those

trends out to a hypothetical 1M data points. The similarity algorithm was best estimated using a polynomial fit, while the MCP2T model was best estimated by a constant, $1x10^{-3}$.
